# Information Geometrical Characterization of Quantum Statistical Models in Quantum Estimation Theory

**DOI:** 10.3390/e21070703

**Published:** 2019-07-18

**Authors:** Jun Suzuki

**Affiliations:** Graduate School of Informatics and Engineering, The University of Electro-Communications, 1-5-1 Chofugaoka, Chofu-shi, Tokyo 182-8585, Japan; junsuzuki@uec.ac.jp

**Keywords:** quantum parameter estimation, quantum Fisher metric, D-invariant model, asymptotically classical model

## Abstract

In this paper, we classify quantum statistical models based on their information geometric properties and the estimation error bound, known as the Holevo bound, into four different classes: classical, quasi-classical, D-invariant, and asymptotically classical models. We then characterize each model by several equivalent conditions and discuss their properties. This result enables us to explore the relationships among these four models as well as reveals the geometrical understanding of quantum statistical models. In particular, we show that each class of model can be identified by comparing quantum Fisher metrics and the properties of the tangent spaces of the quantum statistical model.

## 1. Introduction

Information geometry about a statistical model offers simple and powerful understanding of statistical inference problems (see, for example, books [1,2,3] and the recent review on the subject [4]). A family of probability distributions is regarded as an element of a statistical manifold. Thereby, we can introduce geometrical quantities such as a Riemannian metric and affine connection. The celebrated Chentsov’s theorem selects the unique metric (up to a constant factor), called the Fisher metric or Fisher information matrix, which is invariant under the Markov map. Statistically meaningful connections are also given as a one-parameter family, known as the α-connection, which exhibits the famous duality relationship.

The non-commutative extension of classical statistics to a quantum system was initiated in the 1960s by Helstrom [5] and has been one of the fundamental problems in quantum information theory until today. In particular, recent advances in quantum metrology, quantum sensing, and quantum imaging, i.e., high precision measurement methods utilizing quantum resources, has triggered many activities in the field (see reviews on these subjects [6,7,8,9,10,11]). Despite these efforts in past, there exist many open problems regarding multi-parameter estimation problems.

The information geometrical study on quantum statistical manifolds is of fundamental interest, yet is still at the developing stage [2,4,12,13]. For example, the uniqueness of Fisher metric does no longer holds in the quantum case. Instead, we have a family of operator monotone metrics on quantum statistical manifold [14]. Related to this non-uniqueness of a metric, the asymptotically achievable bound for the mean square error matrix is not expressed as a simple form of a certain quantum version of Fisher metric in general. Recent studies on the first-order asymptotic theory proves that the Holevo bound [15] can be achieved under certain regularity condition [16,17,18,19,20,21]. The Holevo bound, as defined in Section 3.1, is expressed as a nonlinear optimization problem. Hence, it is not totally clear whether we can draw any geometrical structure for it or not. Holevo introduced a particular class of quantum statistical models, called a D-invariant model, based on the invariant property of the tangent space, and showed that the right logarithmic derivative (RLD) Cramér–Rao (CR) bound can be achieved for the D-invariant model. To our knowledge, less is known regarding a connection between information geometrical properties of quantum statistical models and the Holevo bound.

One of the main motivations of this paper is to make an attempt at classifying quantum statistical models based on the Holevo bound, and then to associate these classified models with geometrical quantities. The current paper is based on the results presented in Ref. [22], where we analyzed the structure of the Holevo bound in detail for a qubit system. We derived an explicit formula for any qubit model together with characterization of special classes of the qubit models. We also classified the D-invariant model for the general qubit model together with nontrivial characterization of this model. However, we did not develop a systematic analysis of characterizations for these models in the previous study. What was missing in the previous studies was to consider two different tangent spaces in a unified manner. In this paper, we continue to explore possible classification of arbitrary quantum statistical models into several classes in which the Holevo bound can be expressed in closed formulas. By treating two tangent spaces with equal footings, we derive simple yet geometrically transparent characterizations of statistically meaningful models. Another contribution of this paper is to show that the super-operator, which was originally introduced to study the D-invariant model, also enables us to characterize other classes of quantum statistical models.

**Figure 1:**
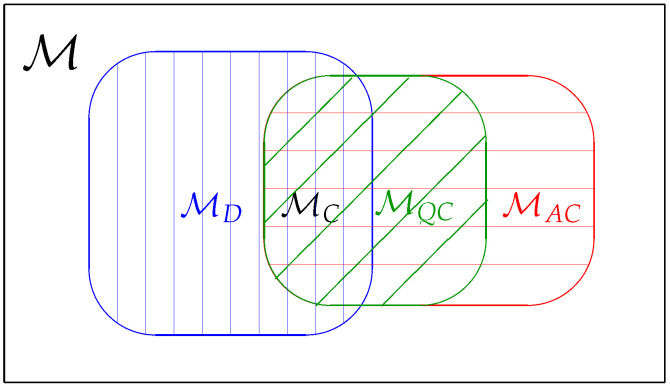
A schematic diagram for model classification of quantum parametric models. A generic quantum parametric model M is indicated by the rectangular box. The blue vertically shadowed area represents the D-invariant model. The red horizontally shadowed area is the asymptotically classical model. The green diagonally shadowed area is the quasi-classical model. The intersection of the D-invariant model and the asymptotically classical model represents the classical model.

**Figure 2:**
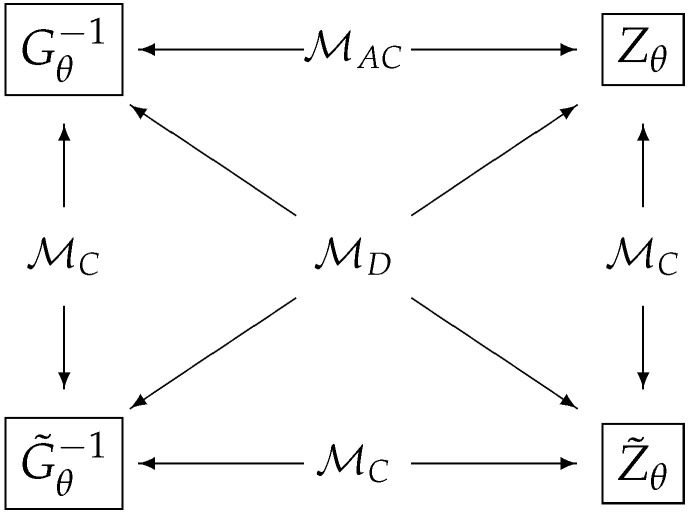
A schematic diagram for model classification for three classes: the classical ($M_{C}$), D-invariant ($M_{D}$), and asymptotically classical ($M_{AC}$) in terms of four matrices $G_{\theta }^{-1},{\tilde{G}}_{\theta }^{-1},Z_{\theta },{\tilde{Z}}_{\theta }$. Two arrows in the opposite direction indicate if two matrices are identical, and the model belongs to a class indicated between these arrows.

In this paper, we consider four different classes: The first class is the classical model where a quantum statistical model is reduced to a statistical model in classical statistics. The second class is known as the quasi-classical model defined by the condition imposing all quantum score operators commute with each other. The third class is known as the D-invariant model introduced by Holevo [15]. It was shown in Ref. [22] that the Holevo bound is equivalent to the RLD CR bound if and only if the model is D-invariant. The fourth class is when the Holevo bound coincides with the symmetric logarithmic derivative (SLD) CR bound. We call this class of models as the asymptotically classical in the sense that the model is asymptotically equivalent to a classical Gaussian model in the local asymptotic normality (LAN) theory [16,17,18,19,20,21]. The results of this paper are given by the propositions and theorem in Section 4.1. In Figure 1, we summarize the relations among four different classes of quantum statistical models. Figure 2 in Section 4.1 represents a schematic diagram for the main theorem of this paper. Out result shows that three classes of models can be characterized simply by comparing quantum Fisher metrics.

The content of this paper is summarized as follows. Section 2 provides preliminaries for notations and mathematical tools used in this paper. In Section 2.3, a few lemmas are proven to be useful for classifying quantum statistical models. In Section 3, we list the definitions of four different classes of statistical models. Our main results are given in the next section. Section 4.1 gives the main results of this paper. In Section 4.2, we discuss the geometrical meaning of the results in detail. Proofs for the theorem and propositions are given in Section 4.3. Several examples are discussed in Section 5 to illustrate our findings. The last section, Section 6, concludes the paper with a few remarks and open problems.

## 2. Preliminaries

A quantum system H is a *d*-dimensional Hilbert space on the complex number. Denote by L(H) a set of (bounded) linear operators from H to itself, and by $L_{h}\left(H\right)$ a set of linear and hermite operators from H to itself. A quantum state is a positive semi-definite operator on H with unit trace. Let us denote a set of all quantum states on H by $\bar{S}\left(H\right)$ and all full-ranked quantum states by S(H). A quantum statistical model is defined by a parametric family of quantum states

(1) $$M:=\{\rho_{\theta }\in S\left(H\right)\;|\;\theta =\left(\theta^{1},\theta^{2},\cdots ,\theta^{n}\right)\in \Theta \},$$

where Θ is an open subset of $R^{n}$. As in classical statistics, we impose several regularity conditions, such as one-to-one smooth mapping, $\theta \mapsto \rho_{\theta }$, differentiability, linearly independence of partial derivatives $\partial \rho_{\theta }/\partial \theta^{i}$ with respect to the coordinates $\left(\theta^{i}\right)$, non-degeneracy for the eigenvalues, and so on. In the following discussions, we assume all these regularity conditions to avoid non-regular behaviors of the statistical model. In particular, we mainly consider a quantum statistical model of full-rank states unless stated explicitly.

### 2.1. Tangent Space and Quantum Fisher Metric

We define two quantum versions of the score functions, called logarithmic derivative operators, as follows. For a given quantum state $\rho_{\theta }$ and any operators X,Y∈L(H), define the symmetric logarithmic derivative (SLD) and right logarithmic derivative (RLD) inner products by

(2) $$\begin{array}{cc} {\langle X,Y\rangle }_{\rho_{\theta }}^{S} & :=\frac{1}{2}\mathrm{tr}\left(\rho_{\theta }\left(YX^{†}+X^{†}Y\right)\right),\\ {\langle X,Y\rangle }_{\rho_{\theta }}^{R} & :=\mathrm{tr}\left(\rho_{\theta }YX^{†}\right), \end{array}$$

respectively, where $X^{†}$ denotes the hermite conjugate of *X*. When *X* and *Y* are both hermite, 〈X,Y〉ρθS=Re〈X,Y〉ρθR holds. The *i*th SLD and RLD operators, $L_{i}$ and ${\tilde{L}}_{i}$, are formally defined by the solutions to the operator equations:

(3) $$\begin{array}{cc} \partial_{i}\rho_{\theta } & =\frac{1}{2}\left(\rho_{\theta }L_{\theta ,i}+L_{\theta ,i}\rho_{\theta }\right),\\ \partial_{i}\rho_{\theta } & =\rho_{\theta }{\tilde{L}}_{\theta ,i}. \end{array}$$

for i=1,2,⋯,n, where $\partial_{i}:=\frac{\partial }{\partial \theta^{i}}$ denotes the partial derivative with respect to $\theta^{i}$. It is not difficult to see that the SLD operators are hermite, whereas RLD operators are not in general.

The SLD and RLD Fisher metrics, or quantum information matrices, are defined by

(4) $$\begin{array}{cc} G_{\theta } & :=\left[g_{\theta ,ij}\right]\mathrm{with}g_{\theta ,ij}:={\langle L_{\theta ,i},L_{\theta ,j}\rangle }_{\rho_{\theta }}^{S},\\ {\tilde{G}}_{\theta } & :=\left[{\tilde{g}}_{\theta ,ij}\right]\mathrm{with}{\tilde{g}}_{\theta ,ij}:={\langle {\tilde{L}}_{\theta ,i},{\tilde{L}}_{\theta ,j}\rangle }_{\rho_{\theta }}^{R}, \end{array}$$

respectively. It is known that the SLD Fisher metric is the smallest and the real part of RLD Fisher metric is the largest operator monotone metrics on the quantum state space [14].

The SLD tangent space is defined by the linear span of SLD operators:

(5) $$T_{\theta }\left(M\right):={\mathrm{span}}_{R}\left\{L_{\theta ,i}\right\}\subset L_{h}\left(H\right),$$

and the RLD tangent space is defined by the linear span of RLD operators with complex coefficients:

(6) $${\tilde{T}}_{\theta }\left(M\right):={\mathrm{span}}_{C}\left\{{\tilde{L}}_{\theta ,i}\right\}\subset L\left(H\right).$$

Let $G_{\theta }^{-1}=\left[g_{\theta }^{ij}\right]$ be the inverse of the SLD Fisher information matrix and ${\tilde{G}}_{\theta }^{-1}=\left[{\tilde{g}}_{\theta }^{ij}\right]$ be the inverse for the RLD Fisher information matrix. It is convenient to introduce the following linear combinations of the logarithmic derivative operators

(7) $$\begin{array}{cc} L_{\theta }^{i} & :=\sum_{j=1}^{n}g_{\theta }^{ji}L_{\theta ,j},\\ {\tilde{L}}_{\theta }^{i} & :=\sum_{j=1}^{n}{\tilde{g}}_{\theta }^{ji}{\tilde{L}}_{\theta ,j}. \end{array}$$

By definitions, $\left\{L_{\theta }^{i}\right\}$ forms a dual basis for the inner product space ${\langle \cdot ,\cdot \rangle }_{\rho_{\theta }}^{S}$; ${\langle L_{\theta }^{i},L_{\theta ,j}\rangle }_{\rho_{\theta }}^{S}=\delta_{j}^{i}$, which we call the SLD dual operator. The same statement holds for the RLD case.

Noting that the SLD and RLD operators are types of exponential representations of the tangent vector $\partial_{i}$, we can show the next lemma.

**Lemma** **1.** *For ∀X∈L(H), and $\forall f\in C^{\infty }\left(R\right)$, the following holds.*

(8) $${\langle f\left(L_{\theta ,i}\right),X\rangle }_{\rho_{\theta }}^{S}={\langle f\left({\tilde{L}}_{\theta ,i}\right),X\rangle }_{\rho_{\theta }}^{R},$$

**Proof.**  We note that the definitions of logarithmic derivative operators give

(9) $${\langle L_{\theta ,i},X\rangle }_{\rho_{\theta }}^{S}={\langle {\tilde{L}}_{\theta ,i},X\rangle }_{\rho_{\theta }}^{R}=\mathrm{tr}\left(\partial_{i}\rho_{\theta }X\right),$$

and repeated applications of this relation proves

(10) $${\langle {\left(L_{\theta ,i}\right)}^{k},X\rangle }_{\rho_{\theta }}^{S}={\langle {\left({\tilde{L}}_{\theta ,i}\right)}^{k},X\rangle }_{\rho_{\theta }}^{R},$$

for any integer power *k*. It is then easy to prove Equation (8).  □

As an application of this lemma, we have alternative expressions for the quantum Fisher information matrices:

(11) $$\begin{array}{cc} g_{\theta }^{ij} & ={\langle {\tilde{L}}_{\theta }^{i},L_{\theta }^{j}\rangle }_{\rho_{\theta }}^{S},\\ {\tilde{g}}_{\theta }^{ij} & ={\langle {\tilde{L}}_{\theta }^{i},L_{\theta }^{j}\rangle }_{\rho_{\theta }}^{R}, \end{array}$$

which follow directly from definitions of $g_{\theta }^{ij}$ and ${\tilde{g}}_{\theta }^{ij}$.

### 2.2. Commutation Operator

For a given quantum statistical model of Equation (1), we define a super-operator $D_{\rho_{\theta }}$ from L(H) to itself, whose action on X∈L(H) is determined by the operator equation:

(12) $$\left[\rho_{\theta }\;,\;X\right]:=\rho_{\theta }X-X\rho_{\theta }=i\rho_{\theta }D_{\rho_{\theta }}\left(X\right)+iD_{\rho_{\theta }}\left(X\right)\rho_{\theta }.$$

The super-operator $D_{\rho_{\theta }}$, called the commutation operator at θ, was introduced by Holevo [15]. By definition, we can check that the super-operator $D_{\rho_{\theta }}$ is linear. Denoting the identity operator *I*, the following relationship holds

(13) $$L_{\theta ,i}=\left(I+iD_{\rho_{\theta }}\right)\left({\tilde{L}}_{\theta ,i}\right),$$

which can be proven by the direct calculation.

The properties useful in our discussion are given in the next lemma.

**Lemma** **2.** *For ∀X,Y∈L(H), the following relations hold.*

(14) $$\begin{array}{cc} {\langle D_{\rho_{\theta }}\left(X\right),Y\rangle }_{\rho_{\theta }}^{S} & =-{\langle X,D_{\rho_{\theta }}\left(Y\right)\rangle }_{\rho_{\theta }}^{S}, \end{array}$$

(15) $$\begin{array}{cc} {\langle D_{\rho_{\theta }}\left(X\right),Y\rangle }_{\rho_{\theta }}^{R} & =-{\langle X,D_{\rho_{\theta }}\left(Y\right)\rangle }_{\rho_{\theta }}^{R}. \end{array}$$

**Proof.**  The first relationship can be proven directly as

$$\begin{array}{cc} 2{\langle D_{\rho_{\theta }}\left(X\right),Y\rangle }_{\rho_{\theta }}^{S} & =\mathrm{tr}\left(\rho_{\theta }\left(D_{\rho_{\theta }}\left(X\right)Y+YD_{\rho_{\theta }}\left(X\right)\right)\right)\\ & =\mathrm{tr}\left((\rho_{\theta }D_{\rho_{\theta }}\left(X\right)+D_{\rho_{\theta }}\left(X\right)\rho_{\theta })Y\right)\\ & =\mathrm{tr}\left(\left(-i\right)\left[\rho_{\theta },X\right]Y\right)\\ & =-\mathrm{tr}\left(\left(-i\right)\left[\rho_{\theta },Y\right]X\right)\\ & =-\mathrm{tr}\left((\rho_{\theta }D_{\rho_{\theta }}\left(Y\right)+D_{\rho_{\theta }}\left(Y\right)\rho_{\theta })X\right)\\ & =-2{\langle X,D_{\rho_{\theta }}\left(Y\right)\rangle }_{\rho_{\theta }}^{S}, \end{array}$$

and Equation (15) can be proven similarly.  □

### 2.3. Basic Lemmas

In this subsection, we list several lemmas that are used in our discussion. We define two hermite matrices, $Z_{\theta },{\tilde{Z}}_{\theta }$ in terms of SLD and RLD dual operators as follows.

(16) $$\begin{array}{cc} Z_{\theta } & :=\left[z_{\theta }^{ij}\right]\;withz_{\theta }^{ij}:={\langle L_{\theta }^{i},L_{\theta }^{j}\rangle }_{\rho_{\theta }}^{R}, \end{array}$$

(17) $$\begin{array}{cc} {\tilde{Z}}_{\theta } & :=\left[{\tilde{z}}_{\theta }^{ij}\right]\;with{\tilde{z}}_{\theta }^{ij}:={\langle {\tilde{L}}_{\theta }^{i},{\tilde{L}}_{\theta }^{j}\rangle }_{\rho_{\theta }}^{S}. \end{array}$$

By definition, they are complex matrices in general. Hermiteness can be checked directly by

(18) $${\left(z_{\theta }^{ij}\right)}^{*}=\mathrm{tr}\left({\left(\rho_{\theta }L_{\theta }^{j}{L_{\theta }^{i}}^{†}\right)}^{†}\right)=\mathrm{tr}\left(\rho_{\theta }L_{\theta }^{i}{L_{\theta }^{j}}^{†}\right)=z_{\theta }^{ji},$$

where $X^{*}$ denotes the complex conjugation of X∈L(H). Hermiteness of the matrix ${\tilde{Z}}_{\theta }$ can be checked similarly. We remark that $Z_{\theta }$ and ${\tilde{Z}}_{\theta }$ are to be used to construct the quantum Fisher metrics, since they define inner products on the tangent space. However, they are not operator monotone metrics in general.

Together with the SLD and RLD Fisher information matrices, we list four matrices for comparison:

(19) $$\begin{array}{cc} G_{\theta }^{-1} & =\left[g_{\theta }^{ij}\right],\;g_{\theta }^{ij}={\langle L_{\theta }^{i},L_{\theta }^{j}\rangle }_{\rho_{\theta }}^{S},\\ {\tilde{G}}_{\theta }^{-1} & =\left[{\tilde{g}}_{\theta }^{ij}\right],\;{\tilde{g}}_{\theta }^{ij}={\langle {\tilde{L}}_{\theta }^{i},{\tilde{L}}_{\theta }^{j}\rangle }_{\rho_{\theta }}^{R},\\ Z_{\theta } & =\left[z_{\theta }^{ij}\right],\;z_{\theta }^{ij}={\langle L_{\theta }^{i},L_{\theta }^{j}\rangle }_{\rho_{\theta }}^{R},\\ {\tilde{Z}}_{\theta } & =\left[{\tilde{z}}_{\theta }^{ij}\right],\;{\tilde{z}}_{\theta }^{ij}={\langle {\tilde{L}}_{\theta }^{i},{\tilde{L}}_{\theta }^{j}\rangle }_{\rho_{\theta }}^{S}. \end{array}$$

By definition, Re(Zθ−1)=Gθ and ReZθ=Gθ−1 hold, where ReX:=(X+X*)/2 denotes the real part of X∈L(H).

First, it is straightforward to see that the operator $L_{\theta }^{i}-{\tilde{L}}_{\theta }^{i}$ enjoys the following property.

**Lemma** **3.** *$L_{\theta }^{i}-{\tilde{L}}_{\theta }^{i}$ is orthogonal to the SLD tangent space $T_{\theta }\left(M\right)$ with respect to ${\langle \cdot ,\cdot \rangle }_{\rho_{\theta }}^{S}$, and is orthogonal to the RLD tangent space ${\tilde{T}}_{\theta }\left(M\right)$ with respect to ${\langle \cdot ,\cdot \rangle }_{\rho_{\theta }}^{R}$.*

**Proof.**  Direct calculation shows

$$\begin{array}{cc} {\langle L_{\theta ,j},L_{\theta }^{i}-{\tilde{L}}_{\theta }^{i}\rangle }_{\rho_{\theta }}^{S} & ={\langle L_{\theta ,j},L_{\theta }^{i}\rangle }_{\rho_{\theta }}^{S}-{\langle L_{\theta ,j},{\tilde{L}}_{\theta }^{i}\rangle }_{\rho_{\theta }}^{S}\\ & ={\langle L_{\theta ,j},L_{\theta }^{i}\rangle }_{\rho_{\theta }}^{S}-{\langle {\tilde{L}}_{\theta ,j},{\tilde{L}}_{\theta }^{i}\rangle }_{\rho_{\theta }}^{R}\\ & =\delta_{j}^{i}-\delta_{j}^{i}=0, \end{array}$$

where Lemma 1 with f(x)=x is used to get the second line.

Orthogonality to the RLD tangent space with respect to the RLD inner product can be proven similarly.  □

The following matrix inequalities between $G_{\theta }$, ${\tilde{G}}_{\theta }$, $Z_{\theta }$, and ${\tilde{Z}}_{\theta }$ are fundamental.

**Lemma** **4.** *Two matrix inequalities,*

(20) $$\begin{array}{cc} Z_{\theta } & \geq {\tilde{G}}_{\theta }^{-1},\\ {\tilde{Z}}_{\theta } & \geq G_{\theta }^{-1}, \end{array}$$

*hold where the necessary and sufficient condition for the equality is the same and given by ∀i, $L_{\theta }^{i}-{\tilde{L}}_{\theta }^{i}=0$.*

**Proof.**  Let $m_{\theta }^{i}:=L_{\theta }^{i}-{\tilde{L}}_{\theta }^{i}$ and define an n×n hermite matrix,

(21) $${\tilde{M}}_{\theta }:=\left[{\langle m_{\theta }^{i},m_{\theta }^{j}\rangle }_{\rho_{\theta }}^{R}\right].$$

The matrix ${\tilde{M}}_{\theta }$ is then positive semi-definite. Using Lemma 3, we can also express matrix elements of ${\tilde{M}}_{\theta }$ as

$$\begin{array}{cc} {\langle m_{\theta }^{i},m_{\theta }^{j}\rangle }_{\rho_{\theta }}^{R} & ={\langle m_{\theta }^{i},L_{\theta }^{j}\rangle }_{\rho_{\theta }}^{R}-{\langle m_{\theta }^{i},{\tilde{L}}_{\theta }^{j}\rangle }_{\rho_{\theta }}^{R}\\ & ={\langle L_{\theta }^{i},L_{\theta }^{j}\rangle }_{\rho_{\theta }}^{R}-{\langle {\tilde{L}}_{\theta }^{i},L_{\theta }^{j}\rangle }_{\rho_{\theta }}^{R}\\ & =z_{\theta }^{ij}-\sum_{k}{\tilde{g}}_{\theta }^{ik}{\langle {\tilde{L}}_{\theta ,k},L_{\theta }^{j}\rangle }_{\rho_{\theta }}^{R}\\ & =z_{\theta }^{ij}-\sum_{k}{\tilde{g}}_{\theta }^{ik}{\langle L_{\theta ,k},L_{\theta }^{j}\rangle }_{\rho_{\theta }}^{S}\\ & =z_{\theta }^{ij}-{\tilde{g}}_{\theta }^{ij}, \end{array}$$

where the second equality is due to Lemma 3. The third equality follows from definition of the RLD dual operator. The fourth equality is due to Lemma 1. Therefore, we show the matrix inequality ${\tilde{M}}_{\theta }=Z_{\theta }-{\tilde{G}}_{\theta }^{-1}\geq 0$. The equality is satisfied if and only if this matrix ${\tilde{M}}_{\theta }$ is zero. This is equivalent to $m_{\theta }^{i}=L_{\theta }^{i}-{\tilde{L}}_{\theta }^{i}=0$ for all i=1,2,⋯,n.

The second inequality can be proven in the same way by starting with another positive semi-definite matrix $M_{\theta }:=\left[{\langle m_{\theta }^{i},m_{\theta }^{j}\rangle }_{\rho_{\theta }}^{S}\right]$.  □

Next, define $m_{\theta ,i}:=L_{\theta ,i}-{\tilde{L}}_{\theta ,i}$ and consider another hermite matrix $M_{\theta }:=\left[{\langle m_{\theta ,i},m_{\theta ,j}\rangle }_{\rho_{\theta }}^{S}\right]$. Following exactly the same logic as in Lemma 4, we can prove the next lemma.

**Lemma** **5.** *Two matrix inequalities*

(22) $$\begin{array}{cc} G_{\theta }+{\tilde{G}}_{\theta }{\tilde{Z}}_{\theta }{\tilde{G}}_{\theta } & \geq 2{\tilde{G}}_{\theta },\\ {\tilde{G}}_{\theta }+G_{\theta }Z_{\theta }G_{\theta } & \geq 2G_{\theta }, \end{array}$$

*hold where the necessary and sufficient condition for the equality is the same and given by ∀i, $L_{\theta ,i}-{\tilde{L}}_{\theta ,i}=0$.*

Finally, the commutation operator and logarithmic operators satisfy the following relations (we note that Equation (35) in the previous publication [22] contains a typo in the same formula, thus Lemma 6 in this paper reports the corrected version). Importantly, the right hand side of each equation is expressed as the difference between two hermite matrices defined in Equation (19).

**Lemma** **6.** (23) 〈Lθi,iDρθ(Lθj)〉ρθS=zθij−gθij=iImzθij,

(24) $$\begin{array}{cc} {\langle {\tilde{L}}_{\theta }^{i},iD_{\rho_{\theta }}\left(L_{\theta }^{j}\right)\rangle }_{\rho_{\theta }}^{S} & ={\tilde{g}}_{\theta }^{ij}-g_{\theta }^{ij}, \end{array}$$

(25) $$\begin{array}{cc} {\langle L_{\theta }^{i},iD_{\rho_{\theta }}\left({\tilde{L}}_{\theta }^{j}\right)\rangle }_{\rho_{\theta }}^{S} & ={\tilde{g}}_{\theta }^{ij}-g_{\theta }^{ij}, \end{array}$$

(26) $$\begin{array}{cc} {\langle {\tilde{L}}_{\theta }^{i},iD_{\rho_{\theta }}\left({\tilde{L}}_{\theta }^{j}\right)\rangle }_{\rho_{\theta }}^{S} & ={\tilde{g}}_{\theta }^{ij}-{\tilde{z}}_{\theta }^{ij}, \end{array}$$

hold for ∀i,j, where ImX:=(X−X*)/2i denotes the imaginary part of X∈L(H).

**Proof.**  Using definitions of the SLD and RLD inner product, and the commutation operator, we have

$$\begin{array}{cc} {\langle X,Y\rangle }_{\rho_{\theta }}^{R}-{\langle X,Y\rangle }_{\rho_{\theta }}^{S} & =\frac{1}{2}\mathrm{tr}\left(\rho_{\theta }\left[Y\;,\;X^{†}\right]\right)\\ & =\frac{1}{2}\mathrm{tr}\left(\left[\rho_{\theta }\;,\;Y\right]X^{†}\right)\\ & =\frac{i}{2}\mathrm{tr}\left(\left(\rho_{\theta }D_{\rho_{\theta }}\left(Y\right)+D_{\rho_{\theta }}\left(Y\right)\rho_{\theta }\right)X^{†}\right)\\ & =\frac{i}{2}\mathrm{tr}\left(\rho_{\theta }\left(D_{\rho_{\theta }}\left(Y\right)X^{†}+X^{†}D_{\rho_{\theta }}\left(Y\right)\right)\right)\\ & ={\langle X,iD_{\rho_{\theta }}\left(Y\right)\rangle }_{\rho_{\theta }}^{S}, \end{array}$$

for all X,Y∈L(H). We now set $X=L_{\theta }^{i},Y=L_{\theta }^{j}$, and then this proves Equation (23). The choice of $X={\tilde{L}}_{\theta }^{i},Y=L_{\theta }^{j}$ and the use of the expressions in Equation (11) gives Equation (24). The relation in Equation (25) can be shown similarly by $X=L_{\theta }^{i}$ and $Y={\tilde{L}}_{\theta }^{j}$. Last, if we let $X={\tilde{L}}_{\theta }^{i},Y={\tilde{L}}_{\theta }^{j}$, we obtain Equation (26).  □

## 3. Model Class in Quantum Statistical Models

In this section, we first define the Holevo bound. We then consider four different classes for quantum statistical models. The first class is purely classical. The second class is a quasi-classical model. The third and fourth ones are nontrivial, the D-invariant and asymptotically classical models, respectively.

### 3.1. Asymptotic Bound: Holevo Bound

In this subsection, we give a brief summary of the asymptotic theory on quantum state estimation [13]. As in classical statistics, we are given *N*-tensor product of identically and independently distributed (i.i.d.) quantum states $\rho_{\theta }^{⊗N}:=\rho_{\theta }⊗\rho_{\theta }⊗\cdots ⊗\rho_{\theta }$ on H. We perform a measurement ${\hat{\Pi }}^{\left(N\right)}$ on $\rho_{\theta }^{⊗N}$, which is described by a set of matrices under certain conditions, to infer an unknown parameter value θ. The estimation error of the measurement ${\hat{\Pi }}^{\left(N\right)}$ is evaluated by the standard mean-square error (MSE) matrix $V_{\theta }^{\left(N\right)}\left[{\hat{\Pi }}^{\left(N\right)}\right]$. In the asymptotic theory of quantum state estimation, one minimizes the weighted trace of the MSE matrix under an additional condition as follows (to distinguish trace tr(·) on the state space, we use Tr{·} for the trace on the parameter space).

(27) $$C_{\theta }\left[W\right]:=\underset{\{{\hat{\Pi }}^{\left(N\right)}\}isa.u.}{\inf}\left\{\underset{N\to \infty }{lim sup}\;N\;\mathrm{Tr}\left\{WV_{\theta }^{\left(N\right)}\left[{\hat{\Pi }}^{\left(N\right)}\right]\right\}\right\},$$

where W>0 is an arbitrary positive-definite weight matrix and a.u. indicates that infimum is taken over all possible asymptotically unbiased estimators. The first-order estimation error bound in Equation (27) is usually referred to as the CR type bound in the literature. There have been many mathematical works to obtain an alternative expression for the CR bound in terms of information quantities, such as the quantum Fisher information matrix. Unlike classical statistics, where the bound is given by the Fisher information matrix, the above bound cannot be written as a simple closed formula in general. However, it takes the following optimization form known as the Holevo bound:

(28) $$C_{\theta }^{H}\left[W\right]:=\underset{\vec{X}\in X_{\theta }}{\inf}h_{\theta }\left[\vec{X}|W\right].$$

In this definition, the set $X_{\theta }$ is defined by

$$X_{\theta }:=\left\{\vec{X}=\left(X^{1},X^{2},\cdots ,X^{n}\right)\;|\;\forall i,\;X^{i}\in L_{h}\left(H\right),\;\forall i,\;\mathrm{tr}\left(\rho_{\theta }X^{i}\right)=0,\;\forall i,j,\;\mathrm{tr}\left(\frac{\partial \rho_{\theta }}{\partial \theta^{i}}X^{j}\right)=\delta_{\;i}^{j}\right\}.$$

Introducing the n×n hermite matrix $H_{\theta }\left[\vec{X}\right]:=\left[{\langle X^{i},X^{j}\rangle }_{\rho_{\theta }}^{R}\right]$, we define the function $h_{\theta }\left[\vec{X}|W\right]$ by

hθ[X→|W]:=TrWReHθ[X→]+Tr|W12ImHθ[X→]W12|,

where $\left|X\right|=\sqrt{X^{†}X}$ denotes the absolute value of a linear operator *X*. The following theorem establishes that the Holevo bound is equal to the CR type bound.

**Theorem** **1.** *For a quantum statistical model satisfying the regularity conditions, $C_{\theta }\left[W\right]=C_{\theta }^{H}\left[W\right]$ holds for all weight matrices.*

Proofs based on different assumptions can be found in Refs. [16,17,18,19,20,21]. The Holevo bound is regarded as unification of previously known bounds [23], such as the SLD and RLD CR bounds:

(29) $$\begin{array}{cc} C_{\theta }^{S}\left[W\right] & :=\mathrm{Tr}\left\{WG_{\theta }^{-1}\right\}, \end{array}$$

(30) CθR[W]:=TrWReG˜θ−1+Tr|W12ImG˜θ−1W12|.

Importantly, the relation $C_{\theta }^{H}\left[W\right]\geq \max\left\{C_{\theta }^{S}\left[W\right],C_{\theta }^{R}\left[W\right]\right\}$ holds for all W>0 [15].

### 3.2. Classical Model

At each point θ∈Θ, the quantum state $\rho_{\theta }$ can be diagonalized with a unitary $U_{\theta }$ as $\rho_{\theta }=U_{\theta }\Lambda_{\theta }U_{\theta }^{-1}$, where the diagonal matrix,

(31) $$\Lambda_{\theta }=\left(\begin{array}{cccc} p_{\theta }\left(1\right) & 0 & \cdots & 0\\ 0 & p_{\theta }\left(2\right) & \cdots & 0\\ \vdots & \vdots & \ddots & \vdots \\ 0 & 0 & \cdots & p_{\theta }\left(d\right) \end{array}\right),$$

lists the eigenvalues of the state $\rho_{\theta }$. By definition, $\forall i,\;p_{\theta }\left(i\right)>0$ and $\sum_{i=1}^{d}p_{\theta }\left(i\right)=1$. In other words, $\Lambda_{\theta }$ can be regarded as an element of P(d):= the set of all (positive) probability distributions on the set {1,2,⋯,d}. When the unitary $U_{\theta }$ is independent of θ for all point in Θ, it is clear that any statistical problem is reduced to the classical one. With this identification, we have the following definition.

**Definition** **1** (Classical statistical model)**.** *For a given quantum statistical model of Equation* (1)*, the model is said classical if the family of quantum states $\rho_{\theta }$ can be diagonalized with a θ-independent unitary U as*

(32) $$\rho_{\theta }=U\Lambda_{\theta }U^{-1},$$

*for all parameter values θ∈Θ.*

### 3.3. Quasi-Classical Model

The second class of quantum statistical models is known in the literature. It is called a quasi-classical model, which was originally introduced in Ref. [24].

**Definition** **2** (Quasi-classical model)**.** *A quantum statistical model of Equation* (1) *is said quasi-classical, if all SLD operators commute with each other at all points θ. That is,*

(33) $$\left[L_{\theta ,i}\;,\;L_{\theta ,j}\right]=0,\;\forall i,j\in \left\{1,2,\cdots ,n\right\},$$

*hold for all parameter values θ∈Θ.*

Clearly, if the model is classical, then it is also quasi-classical. However, the converse statement does not hold in general. A simple counter example is discussed in Section 5.2. It is also easy to see that any one-parameter model is automatically quasi-classical.

An important property of quasi-classical models is that we can diagonalize all SLD operators simultaneously. It is then possible to perform a measurement that saturates the SLD CR bound defined in Equation (29) explicitly. Last, the concept of quasi-classical model makes sense for a particular point θ, if all SLDs at θ commute with each other.

### 3.4. D-Invariant Model

Holevo introduced an important class of quantum statistical models based on the commutation operator $D_{\rho_{\theta }}$ [15].

**Definition** **3** (D-invariant model (Holevo))**.** *A quantum statistical model of Equation* (1) *is called D-invariant at θ, if the SLD tangent space at θ is an invariant subspace of the commutation operator.*

Mathematically, this condition is expressed as $\forall X\in T_{\theta }\left(M\right)$, $D_{\rho_{\theta }}\left(X\right)\in T_{\theta }\left(M\right)$ at θ. For our discussion, we focus on the D-invariant model at all θ (global D-invariance). For (globally) D-invariant models, the Holevo bound can be expressed analytically and coincides with the RLD CR bound [15], i.e., ∀W>0, $C_{\theta }^{H}\left[W\right]=C_{\theta }^{R}\left[W\right]$, and its achievability is discussed in the literature.

### 3.5. Asymptotically Classical Model

The last class of quantum statistical models is when the Holevo bound coincides with the SLD CR bound.

**Definition** **4.** *A quantum statistical model of Equation* (1) *is called asymptotically classical, if the Holevo bound is identical to the SLD CR bound for all positive weight matrices.*

Mathematically, this definition is expressed by the condition: ∀W>0, $C_{\theta }^{H}\left[W\right]=C_{\theta }^{S}\left[W\right]$.

## 4. Model Classification and Characterization

In this section, we give classification of quantum statistical models based on the definitions introduced in Section 3. In the following, we denote the set of all classical models, quasi-classical models, D-invariant models, and asymptotically classical models on H by $M_{C}$, $M_{QC}$, $M_{D}$, and $M_{AC}$, respectively. We first list the results on several equivalent characterization of each model class. Discussions on the results are presented followed by the proofs in Section 4.3.

### 4.1. Results

#### 4.1.1. Classical Model

The following proposition characterizes the classical model.

**Proposition** **1.** *For a given (regular) quantum statistical model of Equation* (1)*, the following conditions are all equivalent.*

*1.*  The model is classical (Definition 2).
*2.*  $\forall X\in T_{\theta }\left(M\right)$, $[X\;,\;\rho_{\theta }]=0$.
*3.*  $\forall X\in {\tilde{T}}_{\theta }\left(M\right)$, $[X\;,\;\rho_{\theta }]=0$.
*4.*  $G_{\theta }={\tilde{G}}_{\theta }$.
*5.*  ∀i, $L_{\theta ,i}={\tilde{L}}_{\theta ,i}$.
*6.*  $D_{\rho_{\theta }}\left(T_{\theta }\left(M\right)\right)=0$.
*7.*  $D_{\rho_{\theta }}\left({\tilde{T}}_{\theta }\left(M\right)\right)=0$.
*8.*  The model is D-invariant and asymptotically classical.

*Here, we remind that all statements are made for the global aspect of the model, i.e., for all points θ∈Θ.*

We note that this result, equivalence between Condition 1 and Condition 4, was also stated in Ref. [25].

#### 4.1.2. D-Invariant Model

In Ref. [22], we derived several equivalent characterizations of the D-invariant model. For readers’ convenience, we summarize them in the following proposition together with the new characterization g.

**Proposition** **2.** *Given a quantum statistical model, the following conditions are equivalent.*

*1.*  *M is D-invariant at θ (Definition 3).*
*2.*  *∀W>0, $C_{\theta }^{H}\left[W\right]=C_{\theta }^{R}\left[W\right]$.*
*3.*  *∀i, Dρθ(Lθi)=∑j(ImZθ)jiLθ,j.*
*4.*  $Z_{\theta }={\tilde{G}}_{\theta }^{-1}$
*5.*  *∀i, $L_{\theta }^{i}={\tilde{L}}_{\theta }^{i}$.*
*6.*  *$\forall X^{i}\in L_{h}\left(H\right)$, $X^{i}-L_{\theta }^{i}⊥\;T_{\theta }\left(M\right)$ with respect to ${\langle \cdot ,\cdot \rangle }_{\rho_{\theta }}^{S}$ ⇒$X^{i}-L_{\theta }^{i}⊥\;T_{\theta }\left(M\right)$ with respect to ${\langle \cdot ,\cdot \rangle }_{\rho_{\theta }}^{R}$.*
*7.*  *${\tilde{T}}_{\theta }\left(M\right)$ is an invariant subspace of the commutation operator $D_{\rho_{\theta }}$.*

The proof for equivalence among the first two conditions, Conditions 1 and 2, was given by Theorem 2.5 of Ref. [22]. The proof for equivalence to other five conditions, Conditions 3–6, was given by Lemma B.1 of Ref. [22].

#### 4.1.3. Asymptotically Classical Model

With this notion of the asymptotically classical model, we have the following result.

**Proposition** **3.** *For a regular quantum statistical model, the following equivalences hold:*

*1.*  *M is asymptotically classical (Definition 4).*
*2.*  *$\exists W_{0}>0$, $C_{\theta }^{H}\left[W_{0}\right]=C_{\theta }^{S}\left[W_{0}\right]$.*
*3.*  *$Z_{\theta }=G_{\theta }^{-1}$.*
*4.*  *ImZθ=0.*
*5.*  *∀i,j, $\mathrm{tr}\left(\rho_{\theta }\left[L_{\theta ,i},L_{\theta ,j}\right]\right)=0$.*

We note that the equivalence among Conditions 1, 3, 4 and 5 were first stated in Ref. [26] without proof. Equivalence between the first and the last conditions (Conditions 1 and 5) was independently proven in Ref. [27], in which the authors named the “compatibility condition”. Condition 2 was suggested by Nagaoka (Private communication (2015)).

#### 4.1.4. $G_{\theta }^{-1},{\tilde{G}}_{\theta }^{-1},Z_{\theta },{\tilde{Z}}_{\theta }$ Matrices

Combining the previous lemmas and theorems with additional analysis, we can obtain another characterizations of quantum statistical models based on the four hermite matrices, $G_{\theta }^{-1},{\tilde{G}}_{\theta }^{-1},Z_{\theta },{\tilde{Z}}_{\theta }$. This is summarized in the next theorem.

**Theorem** **2.** *Given a quantum statistical model, we have the following equivalences.*

*1.*  *M is classical. ⇔ $G_{\theta }^{-1}={\tilde{G}}_{\theta }^{-1}$ ⇔ ${\tilde{G}}_{\theta }^{-1}={\tilde{Z}}_{\theta }$ ⇔ $Z_{\theta }={\tilde{Z}}_{\theta }$*
*2.*  *M is D-invariant. ⇔ ${\tilde{G}}_{\theta }^{-1}=Z_{\theta }$ ⇔ $G_{\theta }^{-1}={\tilde{Z}}_{\theta }$*
*3.*  *M is asymptotically classical. ⇔ $G_{\theta }^{-1}=Z_{\theta }$*

Figure 2 gives a schematic diagram summarizing the relations among the matrices $G_{\theta }^{-1},{\tilde{G}}_{\theta }^{-1},Z_{\theta },{\tilde{Z}}_{\theta }$ and corresponding model classification. We can classify three models simply by comparing these four matrices. Proof of this theorem is given in Section 4.3.4.

As the corollary, we can characterize models by the properties of two tangent spaces $T_{\theta }\left(M\right)$ and ${\tilde{T}}_{\theta }\left(M\right)$.

**Corollary** **1.** *Given a quantum statistical model, we have the following equivalences.*

$$\begin{array}{cc} 1.\;M\;is\;classical. & \Leftrightarrow \;D_{\rho_{\theta }}\left(T_{\theta }\left(M\right)\right)⊥\;{\tilde{T}}_{\theta }\left(M\right)with\;respect\;to{\langle \cdot ,\cdot \rangle }_{\rho_{\theta }}^{S}.\\ & \Leftrightarrow \;D_{\rho_{\theta }}\left({\tilde{T}}_{\theta }\left(M\right)\right)⊥\;{\tilde{T}}_{\theta }\left(M\right)with\;respect\;to{\langle \cdot ,\cdot \rangle }_{\rho_{\theta }}^{S}.\\ & \Leftrightarrow \;D_{\rho_{\theta }}\left({\tilde{T}}_{\theta }\left(M\right)\right)⊥\;T_{\theta }\left(M\right)with\;respect\;to{\langle \cdot ,\cdot \rangle }_{\rho_{\theta }}^{S}.\\ & \Leftrightarrow \;D_{\rho_{\theta }}\left({\tilde{T}}_{\theta }\left(M\right)\right)⊥\;{\tilde{T}}_{\theta }\left(M\right)with\;respect\;to{\langle \cdot ,\cdot \rangle }_{\rho_{\theta }}^{R}. \end{array}$$

$$\begin{array}{cc} 2.\;M\;is\;\mathrm{D-invariant}. & \Leftrightarrow \;\forall i,\;L_{\theta }^{i}-{\tilde{L}}_{\theta }^{i}⊥\;D_{\rho_{\theta }}\left(T_{\theta }\left(M\right)\right)with\;respect\;to{\langle \cdot ,\cdot \rangle }_{\rho_{\theta }}^{S}.\\ & \Leftrightarrow \;\forall i,\;L_{\theta }^{i}-{\tilde{L}}_{\theta }^{i}⊥\;D_{\rho_{\theta }}\left({\tilde{T}}_{\theta }\left(M\right)\right)with\;respect\;to{\langle \cdot ,\cdot \rangle }_{\rho_{\theta }}^{S}. \end{array}$$

$$\begin{array}{cc} 3.\;M\;is\;asymptotically\;classical. & \Leftrightarrow \;D_{\rho_{\theta }}\left(T_{\theta }\left(M\right)\right)⊥\;T_{\theta }\left(M\right)with\;respect\;to{\langle \cdot ,\cdot \rangle }_{\rho_{\theta }}^{S}.\\ & \Leftrightarrow \;D_{\rho_{\theta }}\left(T_{\theta }\left(M\right)\right)⊥\;{\tilde{T}}_{\theta }\left(M\right)with\;respect\;to{\langle \cdot ,\cdot \rangle }_{\rho_{\theta }}^{R}.\\ & \Leftrightarrow \;D_{\rho_{\theta }}\left(T_{\theta }\left(M\right)\right)⊥\;T_{\theta }\left(M\right)with\;respect\;to{\langle \cdot ,\cdot \rangle }_{\rho_{\theta }}^{R}.\\ & \Leftrightarrow \;D_{\rho_{\theta }}\left({\tilde{T}}_{\theta }\left(M\right)\right)⊥\;T_{\theta }\left(M\right)with\;respect\;to{\langle \cdot ,\cdot \rangle }_{\rho_{\theta }}^{R}. \end{array}$$

Proof of this corollary is immediate from Theorem 2 and use of Lemmas 1, 2, and 6.

### 4.2. Discussion on the Results

In this subsection, we discuss the geometrical meaning and statistical consequences of the results: Propositions 1, 2, 3, Theorem 2, and Corollary 1.

#### 4.2.1. Tangent Vector

We first note that Conditions 2 and 3 in Proposition 1 are nothing but the condition in Equation (35) in Section 4.3. This is straightforward to understand if we regard $\partial \rho_{\theta }/\partial \theta^{i}$ as an m-representation of the tangent vector $\partial /\partial \theta^{i}$ and $L_{\theta ,i}$ as an e-representation of it with respect to the SLD Fisher metric. The statement applies for the RLD case.

Next, we can compare Condition 5 in Proposition 1 to the condition for the D-invariant model, Condition 5 of Proposition 2: $L_{\theta }^{i}={\tilde{L}}_{\theta }^{i}$ for all *i*. For the quasi-classical model, we have conditions of $[L_{\theta ,i}\;,\;L_{\theta ,j}]=0$ for all i,j by definition. The asymptotically classical model, on the other hand, only requires commutativity of the SLD operators on the support $\rho_{\theta }$ as indicated by Condition 5 in Proposition 3.

#### 4.2.2. Quantum Fisher Metric

Condition 4 in Proposition 1 states that the SLD and RLD quantum Fisher metrics are identical. If this is the case, in fact, all possible monotone metric on S(H) are identical. In other words, they collapse to the single monotone metric. This is because: (1) the imaginary part of the RLD Fisher metric vanishes; and (2) the SLD Fisher metric is the minimum and the real RLD Fisher metric is the maximum monotone metric (Petz’s theorem) [14]. Due to Theorem 2, this condition is also equivalent to ${\tilde{G}}_{\theta }^{-1}={\tilde{Z}}_{\theta }$ and $Z_{\theta }={\tilde{Z}}_{\theta }$.

Next, let us consider the D-invariant model. Condition 4 of Proposition 2 requires that the real part of the inverse of the RLD Fisher metric is identical to that of the SLD Fisher metric. In addition, the imaginary part of the inverse of the RLD Fisher metric needs to be the same as that of $Z_{\theta }$ matrix. By Theorem 2, its dualistic condition $G_{\theta }^{-1}={\tilde{Z}}_{\theta }$ is shown to be equivalent.

Last, the asymptotically classical model only requires that the imaginary part of the $Z_{\theta }$ matrix (Condition 3 of Proposition 3). From this fact, we can easily understand the reason the classical model is equivalent to the D-invariant and asymptotically classical model.

#### 4.2.3. Tangent Space

As the fundamental objects in information geometry, let us analyze the SLD tangent space $T_{\theta }\left(M\right)$. Condition 6 in Proposition 1 means that the SLD tangent space is in the kernel of the commutation operator $D_{\rho_{\theta }}$, whereas the SLD tangent space is an invariant subspace of the commutation operator for the D-invariant model. Next, the asymptotically classical model is characterized by the condition that $D_{\rho_{\theta }}\left(T_{\theta }\left(M\right)\right)$ and $T_{\theta }\left(M\right)$ are mutually orthogonal subspaces. In other words, the action of the commutation operator takes all elements of the SLD tangent space to the outside of this space.

We split the SLD operator into two parts, namely a classical part and a quantum part, where the latter is defined by the change in a unitary direction. Since the $D_{\rho_{\theta }}$ operator maps the commutation relationship to the anti-commutation relationship as in Equation (12), the quantum part of the SLD operator is expressed in terms of the commutation operator. With more analysis, we can show that the condition for the classical model is equivalent to vanishing of the quantum part of SLD operators (see also the discussion given in Ch. 7 of the book [2]).

#### 4.2.4. Asymptotic Bound

The last equivalent condition, Condition 8 in Proposition 1, is a rather straightforward consequence once we combine all ingredients presented in the lemmas and other equivalent conditions for the classical model. However, the statistical implication of this condition is nontrivial in the sense that we only consider properties of asymptotically achievable bounds. One is the bound for the D-invariant model, and the other is the bound for the asymptotically classical model. Another implication of this equivalence is that there is no genuine quantum statistical model that is both D-invariant and asymptotically classical. In this sense, these two classes of quantum statistical models, the D-invariant and asymptotically classical models, are regarded as two extremal and mutually exclusive models.

### 4.3. Proofs

As stated bove, all conditions in this section are about all parameter values θ unless otherwise stated.

#### 4.3.1. Proof for Proposition 1

**Proof.**  Equivalence to Conditions 2 and 3:

First, we note that the definition of the classical model is equivalent to the commutativity of $\rho_{\theta }$ for all different values θ, that is,

(34) $$\left[\rho_{\theta }\;,\;\rho_{\theta^{\prime }}\right]=0\mathrm{for}\;\mathrm{all}\theta \neq \theta^{\prime }.$$

By the standard matrix analysis, this is equivalent to:

(35) $$\forall i,\;[\frac{\partial }{\partial \theta^{i}}\rho_{\theta }\;,\;\rho_{\theta }]=0.$$

From the definitions of the SLD and RLD operators, we can show that the condition in Equation (35) is equivalent to $[L_{\theta ,i}\;,\;\rho_{\theta }]=0$ for all *i*. This is Condition 2. Similarly, Condition (35) can be converted to $[{\tilde{L}}_{\theta ,i}\;,\;\rho_{\theta }]=0$ for all *i*, which is Condition 3.

Equivalence to Conditions 4 and 5:

If the model is classical, the SLD operator $L_{\theta ,i}$ commutes with the quantum state. Hence, operator formulas in Equation (3) defining the SLD and RLD operators are identical. Since the SLD and RLD operator are uniquely defined, we obtain $L_{\theta ,i}={\tilde{L}}_{\theta ,i}$ for all *i*.

Next, assuming Condition 5, matrices ${\tilde{G}}_{\theta }$ and $Z_{\theta }^{-1}$ are identical. Noting ReZθ−1=Gθ, we get Condition 4.

Last, suppose Condition 4, $G_{\theta }={\tilde{G}}_{\theta }$, then, from Lemma 3, this is possible if and only if ImZθ=0 and $L_{\theta }^{i}={\tilde{L}}_{\theta }^{i}$ for all *i*. Since $g_{\theta ,ij}={\tilde{g}}_{\theta ,ij}$, the latter condition leads to $L_{\theta ,i}={\tilde{L}}_{\theta ,i}$ for all i,j.

Equivalence to Conditions 6 and 7:

Condition 6 is to say that the SLD tangent space is in the kernel of the commutation operator. From definition of the commutation operator and the fact that Xρ+ρX=0 implies X=0 if ρ>0, we have

$$\ker D_{\rho_{\theta }}=\left\{X\in L\left(H\right)\;|\;\left[X,\rho_{\theta }\right]=0\right\}.$$

This then immediately establishes equivalence between Conditions 2 and 6. A similar argument applies for Condition 7.

Equivalence to Condition 8:

When the model is classical, Conditions 4 and 5 give $L_{\theta }^{i}={\tilde{L}}_{\theta }^{i}$ for all *i* (D-invariance). Combining it with $L_{\theta ,i}={\tilde{L}}_{\theta ,i}$ leads to $Z_{\theta }=G_{\theta }^{-1}$. Hence, the definitions for D-invariant and asymptotically classical model are clearly satisfied, if the model is classical. Conversely, suppose that the model is D-invariant, ${\tilde{G}}_{\theta }^{-1}=Z_{\theta }$, and asymptotically classical, $Z_{\theta }=G_{\theta }^{-1}$. Then, it gives Condition 4, $G_{\theta }={\tilde{G}}_{\theta }$.

#### 4.3.2. Proof for Proposition 2

**Proof.**  We only need to prove Condition 7 is equivalent to other conditions. When a model is D-invariant, $L_{\theta }^{i}={\tilde{L}}_{\theta }^{i}$ and $D_{\rho_{\theta }}\left(L_{\theta ,i}\right)\in T_{\theta }\left(M\right)$ hold. Hence, it is straightforward to see ${\tilde{T}}_{\theta }\left(M\right)$ is an invariant subspace. Next, suppose $D_{\rho_{\theta }}\left({\tilde{L}}_{\theta ,i}\right)\in {\tilde{T}}_{\theta }\left(M\right)$, i.e., $\exists c_{i,j},\;D_{\rho_{\theta }}\left({\tilde{L}}_{\theta ,i}\right)=\sum_{j}c_{ji}{\tilde{L}}_{\theta }^{j}$. This then yields $c_{ji}=-i\left(g_{ij}-{\tilde{g}}_{ij}\right)$ by taking the inner product with ${\tilde{L}}_{\theta ,k}$ with respect to the RLD inner product and the use of $L_{\theta ,i}-{\tilde{L}}_{\theta ,i}=iD_{\rho_{\theta }}\left({\tilde{L}}_{\theta ,i}\right)$. This relation is reduced to $L_{\theta ,i}=\sum_{j}g_{ij}{\tilde{L}}_{\theta }^{j}$, which is equivalent to $L_{\theta }^{i}={\tilde{L}}_{\theta }^{i}$.  □

#### 4.3.3. Proof for Proposition 3

**Proof.**  First: The third condition ImZθ=0 implies $\forall W>0,\;C_{\theta }^{H}\left[W\right]=C_{\theta }^{S}\left[W\right]$. This is because of $C_{\theta }^{H}\left[W\right]\geq C_{\theta }^{S}\left[W\right],\forall W>0$ and the direct substitution gives

hθ[L→θ|W]=CθS[W]+Tr|W12ImZθW12|=CθS[W].

Here, ${\vec{L}}_{\theta }=\left(L_{\theta }^{1},L_{\theta }^{2},\cdots ,L_{\theta }^{n}\right)\in X_{\theta }$ is the collection of SLD dual operators. This means the set of SLD dual operators is the optimal achieving the lowest value in the definition of the Holevo bound in Equation (28).

By definition, the first condition obviously implies the second one: $\exists W_{0}>0$, $C_{\theta }^{H}\left[W_{0}\right]=C_{\theta }^{S}\left[W_{0}\right]$.

To show that the existence of a weight matrix $W_{0}$ satisfying $C_{\theta }^{H}\left[W_{0}\right]=C_{\theta }^{S}\left[W_{0}\right]$ implies the vanishing of the imaginary part of the matrix $Z_{\theta }$, we prove the contraposition. That is, if ImZθ≠0, then $C_{\theta }^{H}\left[W\right]>C_{\theta }^{S}\left[W\right]$ holds for all weight matrices *W*. Let us use the following substitution for optimizing the Holevo function:

(36) $$\vec{X}=\left(L_{\theta }^{1},L_{\theta }^{2},\cdots ,L_{\theta }^{n}\right)+\left(K_{\theta }^{1},K_{\theta }^{2},\cdots ,K_{\theta }^{n}\right),$$

where $K_{\theta }^{i}$ (i=1,2,⋯,n) are tangent operators orthogonal to all SLD operators $L_{\theta ,i}$ with respect to the SLD inner product. With this, the Holevo function reads

(37) hθ[X→|W]=CθS[W]+TrWReKθ+Tr|W12Im(Zθ+Kθ)W12|,

where n×n matrix $K_{\theta }=\left[{\langle K_{\theta }^{i},K_{\theta }^{j}\rangle }_{\rho_{\theta }}^{R}\right]$ is hermite. We note that the last two terms:

TrWReKθ+Tr|W12Im(Zθ+Kθ)W12| is strictly positive since it vanishes if and only if ReKθ=0 and Im(Zθ+Kθ)=0 hold. However, these two conditions cannot be satisfied due to the assumption $Z_{\theta }\neq 0$ and the positivity of the matrix $K_{\theta }$. Therefore, we show that if ImZθ≠0, we have $C_{\theta }^{H}\left[W\right]>C_{\theta }^{S}\left[W\right]$ for all W>0. Finally, ImZθ=0⇔∀i,j,trρθ[Lθ,i,Lθ,j]=0 can be shown by elementary algebra. Collecting these arguments proves Proposition 3.  □

#### 4.3.4. Proof for Theorem 2

**Proof.**  Equivalence in Condition 1:

Since $G_{\theta }={\tilde{G}}_{\theta }\;\Leftrightarrow \;G_{\theta }^{-1}={\tilde{G}}_{\theta }^{-1}$, the first equivalence is immediate.

To prove the second equivalence to ${\tilde{G}}_{\theta }^{-1}={\tilde{Z}}_{\theta }$ in Condition 1, let us assume first that a model is classical. Condition 7 of Proposition 1 gives

(38) $$D_{\rho_{\theta }}\left({\tilde{L}}_{\theta }^{i}\right)=0,\;\forall i.$$

Then, Equation (26) of Lemma 6 yields ${\tilde{g}}_{\theta }^{ij}-{\tilde{z}}_{\theta }^{ij}=0$ for all i,j. Conversely, if ${\tilde{G}}_{\theta }^{-1}={\tilde{Z}}_{\theta }$ holds, we have the following equivalence from the first matrix inequality in Lemma 5.

$$\begin{array}{cc} \forall i,\;L_{\theta ,i}={\tilde{L}}_{\theta ,i} & \Leftrightarrow G_{\theta }+{\tilde{G}}_{\theta }{\tilde{Z}}_{\theta }{\tilde{G}}_{\theta }=2{\tilde{G}}_{\theta }\\ & \Leftrightarrow G_{\theta }+{\tilde{G}}_{\theta }{\tilde{G}}_{\theta }^{-1}{\tilde{G}}_{\theta }=2{\tilde{G}}_{\theta }\\ & \Leftrightarrow G_{\theta }={\tilde{G}}_{\theta }. \end{array}$$

This proves the converse part.

The last equivalence to $Z_{\theta }={\tilde{Z}}_{\theta }$ in Condition 1 is proven as follows. A classical model gives this condition is straightforward. Conversely, if this condition is satisfied, the second matrix inequality of Lemma 4 is then expressed as

(39) $$Z_{\theta }\geq G_{\theta }^{-1}.$$

Noting Gθ−1=ReZθ, this inequality concludes ImZθ=0. (Otherwise, the matrix inequality does not hold.) This then shows that the model is asymptotically classical, and we have $G_{\theta }^{-1}=Z_{\theta }={\tilde{Z}}_{\theta }$. The condition $G_{\theta }^{-1}={\tilde{Z}}_{\theta }$ holds if and only if the model is D-invariant from Lemma 4. Therefore, the model is asymptotically classical and D-invariant, i.e., the classical model.

Equivalence in Condition 2:

The first equivalence is already proven in Proposition 3, whose proof is given in Ref. [22]. Here, we note that both conditions can be proven immediately if we use Lemma 4.

Equivalence in Condition 3:

This is proven in Proposition 3.  □

## 5. Examples

### 5.1. Qubit Models

When the dimension of the Hilbert space is two, i.e., a qubit system, we can explicitly work out classification of models [22]. To analyze a given qubit model, it is convenient to use the Bloch vector representation of qubit states (see, for example, [15]). Define a three-dimensional real vector $s_{\theta }=\left(s_{\theta }^{i}\right)$ for i=1,2,3 by

(40) $$s_{\theta }^{i}:=\mathrm{tr}\left(\rho_{\theta }\sigma_{i}\right),$$

where $\sigma_{i}$ are the standard Pauli matrices. Since the mapping $s_{\theta }\mapsto \rho_{\theta }$ is bijective, a quantum statistical model for the qubit case can be defined as

(41) $$M=\{s_{\theta }\in R^{3}\;|\;\theta \in \Theta \}.$$

The positivity condition $\rho_{\theta }>0$ is equivalent to $|s_{\theta }|<1$. Based on the Bloch vector $s_{\theta }$, we can derive closed formulas for the quantum score functions (SLD and RLD logarithmic derivative operators) and the quantum Fisher information matrices (see, for example, [22]) In Ref. [22], we derived the following conditions for a model of Equation (41) to be the D-invariant and asymptotically classical.

1. M is D-invariant. ⇔$|s_{\theta }|$ is independent of θ.
2. M is asymptotically classical. ⇔$\partial_{i}s_{\theta }\times \partial_{j}s_{\theta }$ (∀i≠j) is orthogonal to $s_{\theta }$.

The equivalent condition for the D-invariant model immediately tells us that any unitary model on the qubit system is D-invariant. The converse statement is, of course, not true in general. For example, the following two-parameter qubit model is D-invariant, but not unitary.

(42) $$M=\{s_{\theta }=\left(\theta^{1},\theta^{2},\sqrt{s_{0}^{2}-{\left(\theta^{1}\right)}^{2}-{\left(\theta^{2}\right)}^{2}}\right)\;|\;\theta \in \Theta \},$$

where $s_{0}\in \left(0,1\right)$ is a fixed constant and the parameters takes values within the region $\Theta \subset R^{2}$ satisfying the positivity condition for the state.

Next, we can work out whether or not there exists a classical qubit model. It is straightforward to show that there cannot be any multi-parameter classical qubit model under the regularity condition, and thus only one-parameter classical model exists. The reason is simply because there can be a single parameter classical model embedded in a 2×2 matrix space. Any multi-parameter classical model becomes a non-regular model.

Finally, we ask if there exists a quasi-classical model in a qubit system. It turns out that there exists no such a quasi-classical qubit model. This is because imposing commutativity between the SLD operators leads to a non-regular model.

To prove this statement, we note the commutation condition for the SLD operators is expressed in terms of the Bloch vectors as

(43) $$\left[L_{\theta ,i}\;,\;L_{\theta ,j}\right]=0\;\Leftrightarrow \;\partial_{i}s_{\theta }\times \partial_{j}s_{\theta }=0.$$

Consider a two-parameter qubit model. The condition $\partial_{1}s_{\theta }\times \partial_{2}s_{\theta }=0$ is equivalent to linearly dependence of two vectors $\partial_{1}s_{\theta }$, $\partial_{2}s_{\theta }$. This then implies the existence of a function c:Θ→R such that $L_{\theta ,1}=c\left(\theta \right)L_{\theta ,2}$ holds. This contradicts with linearly independence of the tangent vectors. Note that, if this is the case, the dimension of the tangent space is one rather than two. The case of three-parameter models can be checked similarly.

### 5.2. Non-Classical Quasi-Classical Model

As mentioned in Section 3.3, there exists a quantum statistical model that is quasi-classical (all SLD operators commute with each other) and yet non-classical. It is straightforward to observe that such cases arise if a model is non-regular. For example, quantum states are not full rank. Below, we give a simple regular statistical model in a three-dimensional quantum system (d=3).

We consider the following two-parameter model:

$$M:=\{\rho_{\theta }\;|\;\theta =\left(\theta^{1},\theta^{2}\right)\in \Theta \},$$

where

(44) $$\begin{array}{cc} \rho_{\theta } & :=U_{\theta^{2}}\Lambda_{\theta^{1}}U_{\theta^{2}}^{-1}, \end{array}$$

(45) $$\begin{array}{cc} \Lambda_{\theta^{1}} & :=\left(\begin{array}{ccc} \lambda (\theta^{1}) & 0 & 0\\ 0 & c\lambda (\theta^{1}) & 0\\ 0 & 0 & 1-\left(1+c\right)\lambda \left(\theta^{1}\right) \end{array}\right), \end{array}$$

(46) $$\begin{array}{cc} U_{\theta^{2}} & :=\;e^{i\theta^{2}\sigma_{1}}with\sigma_{1}=\left(\begin{array}{ccc} 0 & 1 & 0\\ 1 & 0 & 0\\ 0 & 0 & 0 \end{array}\right), \end{array}$$

with the constant c∈R (c≠1) and smooth function $\lambda (\theta^{1})$ being chosen arbitrary as long as the corresponding classical model for $\Lambda_{\theta^{1}}$

$$M_{1}:=\left\{p_{\theta^{1}}=\left(\lambda \left(\theta^{1}\right),c\lambda \left(\theta^{1}\right),1-\left(1+c\right)\lambda \left(\theta^{1}\right)\right)|\theta^{1}\in \Theta_{1}\right\},$$

satisfies $M_{1}\in P\left(3\right)$. The SLD operators are calculated as

(47) $$\begin{array}{cc} L_{\theta ,1} & =U_{\theta^{2}}\;\frac{\dot{\lambda }}{\lambda }\left(\begin{array}{ccc} 1 & 0 & 0\\ 0 & 1 & 0\\ 0 & 0 & -m(\theta^{1}) \end{array}\right)U_{\theta^{2}}^{-1} \end{array}$$

(48) $$\begin{array}{cc} L_{\theta ,2} & =U_{\theta^{2}}\;2\frac{1-c}{1+c}\left(\begin{array}{ccc} 0 & -i & 0\\ i & 0 & 0\\ 0 & 0 & 0 \end{array}\right)U_{\theta^{2}}^{-1}, \end{array}$$

where $\dot{\lambda }=d\lambda \left(\theta^{1}\right)/d\theta^{1}$, $m\left(\theta^{1}\right)=1-{\left(1-\left(1+c\right)\lambda \left(\theta^{1}\right)\right)}^{-1}$. To have a regular quantum model, we also impose $\dot{\lambda }\neq 0$ for all $\theta^{1}$. It is clear that two SLD operators commute with each other for all θ. The RLD operators are

(49) $$\begin{array}{cc} {\tilde{L}}_{\theta ,1} & =L_{\theta ,1} \end{array}$$

(50) $$\begin{array}{cc} {\tilde{L}}_{\theta ,2} & =U_{\theta^{2}}\;\left(\begin{array}{ccc} 0 & -i(1-c) & 0\\ i(1-c)/c & 0 & 0\\ 0 & 0 & 0 \end{array}\right)U_{\theta^{2}}^{-1}. \end{array}$$

We can show that the SLD Fisher information matrix is diagonal and is given by

(51) $$\begin{array}{cc} g_{\theta ,11} & =\frac{\dot{\lambda }}{\lambda }\left(2+m{\left(\theta^{1}\right)}^{2}\right), \end{array}$$

(52) $$\begin{array}{cc} g_{\theta ,12} & =g_{\theta ,21}=0, \end{array}$$

(53) $$\begin{array}{cc} g_{\theta ,22} & ={\left(2\;\frac{1-c}{1+c}\right)}^{2}\lambda \left(\theta^{1}\right), \end{array}$$

whereas the RLD Fisher information matrix is

(54) $$\begin{array}{cc} {\tilde{g}}_{\theta ,11} & =g_{\theta ,11}, \end{array}$$

(55) $$\begin{array}{cc} {\tilde{g}}_{\theta ,12} & ={\tilde{g}}_{\theta ,21}=0, \end{array}$$

(56) $$\begin{array}{cc} {\tilde{g}}_{\theta ,22} & =\frac{{\left(1-c\right)}^{2}\left(1+c\right)}{c}\lambda \left(\theta^{1}\right). \end{array}$$

It is easy to see that ${\tilde{g}}_{\theta ,22}\geq g_{\theta ,22}$ with equality if and only if c=1, which is excluded. Therefore, $G_{\theta }\neq {\tilde{G}}_{\theta }$ holds and this model is not classical by Proposition 1.

## 6. Concluding Remarks

We derive several equivalent characterizations of quantum statistical models based on the estimation error bound, the Holevo bound, and information geometric properties. This then yields a simple classification of quantum statistical models by calculating information matrices. Our results immediately provide practical advantages for classifying important classes of quantum statistical models in quantum metrology [6,7,8,9,10,11]. Three classes are mainly discussed in this paper: the classical model, D-invariant model, and asymptotically classical model. We also give relationships among these classes. In particular, the classical model can be viewed as the intersection of the D-invariant and asymptotically classical models. These three models have different interpretations based on the information geometrical point of view.

Before closing the paper, we list two open questions to be addressed. In this paper, we focus on the global aspects of the quantum statistical models only. The first extension is then to analyze local properties of each class of the quantum statistical model. In Ref. [22], we analyzed the local properties for the D-invariant and asymptotically classical models. Therefore, it is interesting to see whether the local classical model is a useful concept or not. Second, we only use two quantum Fisher metrics, the SLD and RLD Fisher metrics, together with their variants $Z_{\theta }$ and ${\tilde{Z}}_{\theta }$. We expect that other families of quantum Fisher metrics should also give model classification and characterization.
